# CONSMI: Contrastive Learning in the Simplified Molecular Input Line Entry System Helps Generate Better Molecules

**DOI:** 10.3390/molecules29020495

**Published:** 2024-01-19

**Authors:** Ying Qian, Minghua Shi, Qian Zhang

**Affiliations:** School of Computer Science and Technology, Shanghai Frontiers Science Center of Molecule Intelligent Syntheses, East China Normal University, 3663 North Zhongshan Road, Putuo District, Shanghai 200062, China; yqian@cs.ecnu.edu.cn (Y.Q.); 51215901061@stu.ecnu.edu.cn (M.S.)

**Keywords:** deep learning, drug design, contrastive learning

## Abstract

In recent years, the application of deep learning in molecular de novo design has gained significant attention. One successful approach involves using SMILES representations of molecules and treating the generation task as a text generation problem, yielding promising results. However, the generation of more effective and novel molecules remains a key research area. Due to the fact that a molecule can have multiple SMILES representations, it is not sufficient to consider only one of them for molecular generation. To make up for this deficiency, and also motivated by the advancements in contrastive learning in natural language processing, we propose a contrastive learning framework called CONSMI to learn more comprehensive SMILES representations. This framework leverages different SMILES representations of the same molecule as positive examples and other SMILES representations as negative examples for contrastive learning. The experimental results of generation tasks demonstrate that CONSMI significantly enhances the novelty of generated molecules while maintaining a high validity. Moreover, the generated molecules have similar chemical properties compared to the original dataset. Additionally, we find that CONSMI can achieve favorable results in classifier tasks, such as the compound–protein interaction task.

## 1. Introduction

Discovering new drugs and material molecules can bring tremendous social and technological progress. In particular, for some diseases that do not yet have effective treatment plans, new targeted drugs represent great hope. Discovering more drugs also provides a way to achieve personalized precision medicine [1]. However, drug discovery is a costly, time-consuming process with a high failure rate [2]. The estimated number of potential drug-like candidates ranges from $10^{23}$ to $10^{60}$ molecules [3], but only around $10^{8}$ molecules have been synthesized and investigated so far [4]. In molecular research, researchers are employing a synergy of genetic algorithms and object generation techniques for molecular screening [5,6,7,8]. These methods generally perform Pareto optimization on molecules to generate molecules with ideal properties. Additionally, they are utilizing deep learning generative models for a more accurate simulation of molecular distributions, showcasing the growing reliance on advanced artificial intelligence techniques in molecular research. Firstly, a generation model is used to simulate the molecular distribution using self-supervised learning, and then a series of molecules is generated using auto-regression starting from carbon atoms. With the thriving development of deep learning models in computer vision [9] and natural language processing (NLP) [10], these models are also employed to improve molecular distribution and, when combined with reinforcement learning and multimodal techniques, generate molecules with specific properties. There are two main ways to generate molecules: using graph-based methods and using SMILES notation methods. In addition, molecular generation methods based on SELFIES [11] have been very popular recently.

There is a lot of research on the graph-based method to generate molecules [12,13,14]. This method represents molecules as graph structures, where the nodes in the graph represent atoms and the edges represent chemical bonds. This representation is intuitive and can clearly present the atoms in the molecule and their connection relationships. MOLDR [14] decomposes the molecular graphs in the training dataset into subgraphs and reassembles them in different ways to generate new, optimized molecular graphs. The Junction Tree VAE (JT-VAE) [13] offers an alternative approach to molecular generation by representing molecules as graph tree structures. This representation allows for a more expressive and structured encoding of molecular compounds. One notable advantage of JT-VAE is its ability to guarantee the 100% validity of generated molecules. It achieves this by maintaining a vocabulary of molecular components that can be added at each junction of the molecule tree. By constructing molecules in a step-wise manner based on valid molecular components, JT-VAE ensures that every generated molecule adheres to the predefined rules and constraints of molecular validity. This design has three key limitations. Firstly, when using JT-VAE for attribute optimization, the task becomes more challenging because two molecules with the same connection tree may correspond to significantly different attributes. Secondly, the absence of consideration of the node order arrangement during the generation process can result in increased time consumption. Thirdly, due to the complexity of real-world drug molecules, generating substructures with less than 20 atoms is impractical [15].

The Simplified Molecular Input Line Entry System (SMILES) notation [16], which represents molecules as character strings, allows for the application of advanced deep learning models from the field of NLP for computational tasks involving molecules [17]. By treating molecules as sequences of characters, NLP models can be leveraged to analyze and generate molecular structures, enabling the exploration of chemical space using techniques inspired by language processing [18]. In the initial development of deep learning architectures for molecular generation, recurrent neural networks (RNNs) [19] were widely used with molecular SMILES representations [20,21]. These models were trained on extensive datasets of molecules and further refined through reinforcement learning [22,23] or transfer learning methods [21]. The goal was to generate molecules with specific properties and activities by guiding the model towards desired outcomes. These early approaches played a crucial role in advancing the field of deep learning for molecular generation and paved the way for subsequent research in the area. AE-based models have also played a significant role in molecular generation tasks used with SMILES. The molecule generative model based on VAE can generate various molecules with the required properties by learning the potential space following a specific probability distribution [24,25]. The AAE (adversarial auto-encoder) [26], on the other hand, incorporates adversarial training principles. By introducing a discriminator network, the AAE encourages the encoder to produce latent representations that closely resembled the true SMILES distribution [27]. This adversarial training improved the quality and diversity of generated molecules. generative adversarial networks (GANs) [28] have emerged as another popular approach for molecular design and generation used with SMILES representation. ORGANs (objective-reinforced generative adversarial networks) [29], as an early method of using the GAN model for molecular generation, guide the process of generation through the gradual construction process of generating molecules and through the use of reinforcement learning. However, the chemical feasibility of ORGAN-generated molecules is very low. LatentGAN [30] is a model that combines both an auto-encoder and a generative adversarial network for molecular generation. It starts by pre-training an auto-encoder using SMILES structures as input, and then trains a GAN to generate latent vectors for corresponding molecules. Similarly, LatentGAN faces the problem of poor validity and diversity of generated molecules.

Self-Referencing Embedded Strings (SELFIES) [11] represents a significant advancement in the field of molecular string representations, designed to address some of the limitations inherent in the traditional SMILES format. Compared to SMILES, SELFIES separates the information of branches and loops, improving the robustness of syntax. This robustness ensures that every generated string corresponds to a valid molecular structure, greatly benefiting machine learning applications in chemistry. This advancement is particularly notable in the context of machine learning applications for molecular generation. PASITHEA [31] is a SELFIES-based model for generating molecular structures, leveraging direct-gradient-based optimization techniques from computer vision. Researchers from the University of Toronto have developed STONED [32], a simple and efficient generative model that does not require training. This model is capable of enumerating structures and searching for transformative trajectories between molecules within a localized chemical space. SELFIES also has limitations, including potentially complex and less readable strings and higher computational demands for encoding and decoding processes.

Recently, there has been a lot of research on using the transformer [10] model to perform molecular generation tasks based on SMILES [33,34,35]. The transformer architecture comprises an encoder and a decoder for sequence tasks. It utilizes self-attention to capture dependencies and employs feed-forward networks. This design has powered advancements in natural language processing tasks like translation and generation. Generally speaking, researchers use a transformer encoder to encode molecule-related information, such as the three-dimensional structural information of molecules [34], molecule-related proteins [33], etc., and use a decoder to generate a SMILES. Of course, there are also cases where an encoder (Bert) is used alone for supervised learning tasks such as compound–protein interaction classification [36,37] or a decoder (GPT) is used alone for unconditional molecular generation [35]. MolGPT [35] has achieved high effectiveness in unconditional molecular generation, but its novelty is low, indicating severe overfitting of the model.

Besides enhancing the generation model, researchers are also exploring ways to more effectively use data. There are several methods to augment data for molecular generative models, and one of the most common approaches is SMILES enumeration [38]. SMILES enumeration means that a molecule can have multiple valid SMILES representations. Multiple SMILES representations of a molecule represent more comprehensive structural information of the molecule. Josep et al. [39] used randomized SMILES to expand data, effectively improving the molecular generative model compared to using canonical SMILES. Cheng-Kun Wu [40] also used SMILES enumeration to expand the dataset to improve the effectiveness of latent representation learning from molecules. However, the current SMILES enumeration method is only used for simple dataset expansion and has not been appropriately optimized for the concrete model.

In addition to simple data augmentation, researchers have recently used contrastive learning to learn better molecular representations for downstream tasks of molecules. The core of contrastive learning lies in constructing positive and negative sample pairs and utilizes the normalized temperature-scaled cross-entropy loss (NT-Xent) [41] to encourage the model to learn meaningful representations by maximizing agreement between positive pairs (augmented versions of the same image) and minimizing agreement between negative pairs (augmented versions of different images). Gathering positive instances typically encompasses various enhanced perspectives of identical data (such as data augmentation), while negative pairs commonly consist of the remaining samples within the mini-batch. Thus, the key to this matter is how to effectively enhance data. In the field of computer vision, approaches like SimCLR, proposed by Chen et al. [42], used image cropping, rotation, and other methods to enhance the data. In the textual domain, approaches like [43] utilize back-translation to enhance the data [44]. SimCSE [45] enters a sample into the model twice and then puts it through dropout twice, obtaining two different views that are mutually positive samples. In the molecular field, approaches like MolCLR [46] encompasses three molecular graph enhancement strategies for data enhancement: atomic masking, key deletion, and subgraph deletion. MoCL [47] combines the basis of ordinary graph data enhancement and domain knowledge to ensure that the representation of molecular graphs does not change during the enhancement process. SMICLR [48] uses different representations of molecules (SMILES representation and graph representation methods) as data augmentation for molecules.

We proposed a framework called CONSMI that utilizes the SMILES enumeration strategy as a data augmentation strategy for contrastive learning, and the trained representations achieved good results in self-supervised molecular generation and supervised compound–protein interaction prediction experiments. The molecular generation model is based on GPT, which effectively solves the problem of the overfitting of GPT-generated molecules compared to MolGPT [35]. The compound protein interaction model is based on a unified transformer and has achieved SOTA results on multiple datasets.

The contributions of this article are as follows:

1. We propose CONSMI, a contrastive learning framework that learns representation from a large molecular dataset.
2. The CONSMI framework combined with a transformer decoder generates more successful molecules.
3. The CONSMI framework combined with a transformer encoder achieves SOTA results on multiple datasets of compound–protein interactions.

## 2. Results and Discussion

In this section, we first demonstrate the performance of the molecular generation task. We compared the model with other state-of-the-art (SOTA) methods and conducted some interpretability analyses. Then, we demonstrated the performance of the model on some classification tasks. We refer to the GPT model with the CONSMI framework’s pre-trained CONSMI embedding layer as CON-GPT, and indicate the two fine-tuning methods (frozen and unfrozen) after the model name. The models for comparative experiments have been introduced in Introduction.

### 2.1. Molecular Generation Results

As mentioned before, a good molecular generative model needs to generate more valid, unique, and novel molecules. Therefore, CON-GPT is evaluated in comparison to previous approaches using these evaluation criteria. Notably, JT-VAE utilizes graph representations as input, while the other approaches utilize SMILES.

The results on the Moses dataset are shown in Table 1. We conducted comparative experiments using the methods mentioned in the introduction: CharRNN, VAE, AAE, LatentGAN, JT-VAE, and MolGPT. Due to the fact that JT-VAE performs verification at every step of molecule generation, the validity of the model is 1. With the exception of JT-VAE, the MolGPT model achieves the highest validity score of 0.995 for molecule generation. However, its novelty score is only 0.781, indicating significant overfitting. It is important to note that both the validity and novelty of generated molecules are crucial. The CON-GPT model, whose SMILES embedding is pre-trained using the contrastive learning approach with the CONSMI framework, exhibits a validity score that is 0.04 lower compared to the MolGPT model. However, it achieves a higher novelty score of 0.834, indicating more valid and novel generated molecules. We found that the $IntDiv_{1}$ of the baseline method fluctuates around 0.855, while ours is around 0.850. Although the difference is not obvious, we found that GPT-based methods all have this problem, which is a point that we need to study in the future. The success rate of our CON-GPT exceeds that of all methods except JT-VAE and LatentGAN. LatentGAN has drawbacks related to GAN, such as unstable training and time consumption. This suggests that the CONSMI framework effectively learns the diversity of SMILES grammar, mitigates the overfitting issue observed in the MolGPT model, and provides a more improved deep molecular generative model based on SMILES.

We conducted experiments to compare two methods of fine-tuning the models: frozen (freezing the pre-training CONSMI embedding weights) and unfrozen (unfreezing the pre-training CONSMI embedding weights). We found that the freezing method outperforms the unfreezing method, despite our initial expectations. The CON-GPT model with unfrozen pre-training weights showed a slight decrease in validity (of 0.004) compared to the MolGPT model, but exhibited a slight increase in novelty (of 0.01). On the other hand, the CON-GPT model with frozen pre-training weights also experienced a decrease in validity (of 0.005) but showed a significant increase in novelty (of 0.053). Additionally, freezing the pre-training weights led to faster model training. These results demonstrate the strong feature extraction and generalization capabilities of our CONSMI framework.

In order to evaluate the generalization ability of our model, we conducted experiments on the GuacaMol dataset, which is a subset of the ChEMB dataset. The results on the GuacaMol dataset are shown in Table 2. It is worth noting that the pre-trained dataset and MOSES dataset used in our experiments are both subsets of the ZINC dataset. By assessing our model’s performance on the GuacaMol dataset, we can learn how well it can generate molecules with desirable properties beyond the datasets it was specifically trained on. This evaluation provides valuable insights into the model’s ability to generalize and produce high-quality molecules in diverse chemical spaces.

The experimental results on the GuacaMol dataset exhibit similarities to those obtained on the MOSES dataset. The GPT model enhanced with the CONSMI framework for pre-training demonstrates a higher uniqueness and novelty compared to the pure GPT model, albeit at the cost of a slight decrease in validity. The performance difference between the fine-tuning methods for unfrozen and frozen weights aligns with the observations on the MOSES dataset. Overall, there is still a slight advantage in generating valid and novel molecules, indicating a certain degree of generalization ability in our model. However, the leading advantage is not as pronounced as on MOSES, and we speculate that this may be due to the different sources of the GuacaMol dataset and our pre-trained dataset.

**Figure 1:**
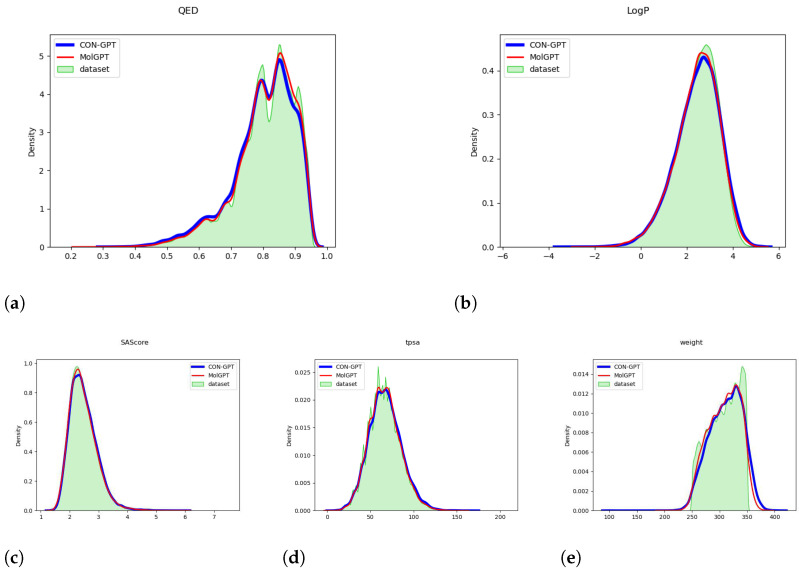
The distribution of molecular attributes generated by the model trained on the MOSES dataset. (**a**) QED (Quantitative Estimate of Drug-likeness). (**b**) LogP (Octanol-Water Partition Coefficient). (**c**) SAScore (Synthetic Accessibility Score). (**d**) TPSA (Topological Polar Surface Area). (**e**) Weight (Molecular Weight).

**Figure 2:**
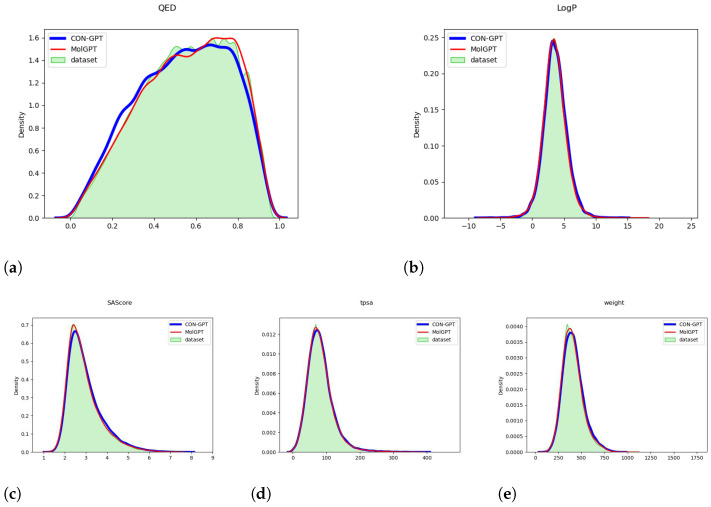
The distribution of molecular attributes generated by the model trained on the GuacaMol dataset. (**a**) QED (Quantitative Estimate of Drug-likeness). (**b**) LogP (Octanol-Water Partition Coefficient). (**c**) SAScore (Synthetic Accessibility Score). (**d**) TPSA (Topological Polar Surface Area). (**e**) Weight (Molecular Weight).

Figure 1 and Figure 2 provide compelling evidence that the important molecular attributes (QED, LogP, SAScore, tpsa, weight) generated by the model closely match the distribution of the original dataset. These data were calculated by the RDKit library [49]. This result strongly suggests that the model has successfully learned the underlying distribution of molecular attributes present in the dataset.

**Case Study** We systematically examined all generated molecules with QED values exceeding 0.9 and assessed their similarity to the molecules in the Moses test set. As shown in Table 3 and Table 4, We found that although the model has not seen the molecules in the test set, a substantial number of the generated molecules exhibited a similarity of over 0.9 to those in the test set.
**Adjusting the Temperature** We conducted an evaluation to understand how adjustments to the hyperparameter τ impact NT-Xent’s ability to distinguish effectively between positive and negative samples. This assessment involved exploring a set of τ values that are frequently used in practice, specifically 0.05, 0.1, 0.5, and 1, to identify the most suitable τ value. The results, encapsulated in Table 5, display the validation errors corresponding to each τ value during the model’s training process. We found that a τ of 0.1 yielded the most favorable results. Intriguingly, these results corroborate well with the existing literature, particularly concerning applications in molecular data [48].

### 2.2. Compound–Protein Interaction Results

In this section, we assessed the performance of the CONSMI architecture in the context of compound–protein interactions. In simpler terms, the task can be boiled down to a binary classification problem: determining whether there is an interaction between molecules and proteins or not. We used a transformer with two encoders and denoted our baseline model as UniT [50]. We substituted the original SMILES embedding layer with the trained CONSMI embedding Layer, referred to this as CON-UniT. We conducted comparisons between our model and some currently popular or highly effective models, such as GNN-CPI [51], TransformerCPI [36], Moltrans [37], and BCM-DTI [52]. GNN-CPI and TransformerCPI both utilize molecular graph representations of drugs and employ a CNN and a GCN (graph convolutional network), respectively, to encode the graphs. TransformerCPI and Moltrans, on the other hand, utilize transformer encoders to capture the chemical semantics. Additionally, Moltrans is a substructure-based DTI (drug–target interaction) prediction approach that applies byte pair encoding (BPE) to decompose drug and protein sequences into a set of explicit substructure sequences [52].

Table 6 and Table 7 illustrate the notable performance improvements achieved by the UniT model with pre-training in compound–protein interaction classification tasks. The model exhibits a significantly higher F1 value, accuracy, and recall compared to the model without pre-training. Compared to the currently popular frameworks, our model excels in all aspects except for recall, where it does not always achieve the highest performance. However, it outperforms other models in terms of precision and demonstrates the remarkable ability to maintain a balance between precision and recall, even on the imbalanced DAVIS dataset [53]. This demonstrates the effectiveness of the pre-training approach in enhancing the model’s ability to accurately classify compound–protein interactions. In addition, as seen in the table, a stand-alone transformer model does not necessarily outperform other models in terms of precision. However, the CON-UniT model demonstrates a significant competitive advantage. It is worth mentioning that the other models have undergone specific adjustments for the compound–protein interaction task, whereas our model achieved such impressive results with only the addition of pre-training to the original UniT. This observation further indicates that our CONSMI framework successfully learns meaningful representations of molecules. By leveraging the contrastive learning framework, our model is able to capture important features in the molecular data, leading to an improved performance in compound–protein interaction classification tasks. The effectiveness of the CONSMI framework highlights its ability to enhance the model’s understanding and representation of molecular structures, thereby facilitating better predictions and classification accuracy.

## 3. Methods

Here, we describe the method proposed in this work. We propose a contrastive learning pre-trained framework called CONSMI, which consists of a CONSMI embedding layer, a transformer encoder layer, and a projection head. We used the pre-trained CONSMI embedding layer for the molecular generation model CON-GPT. In order to demonstrate universality, we also used the CONSMI embedding layer for the classification model CON-UniT. CON-GPT is based on a transformer decoder, while CON-UniT is based on a transformer encoder.

### 3.1. CONSMI Framework

**Figure 3:**
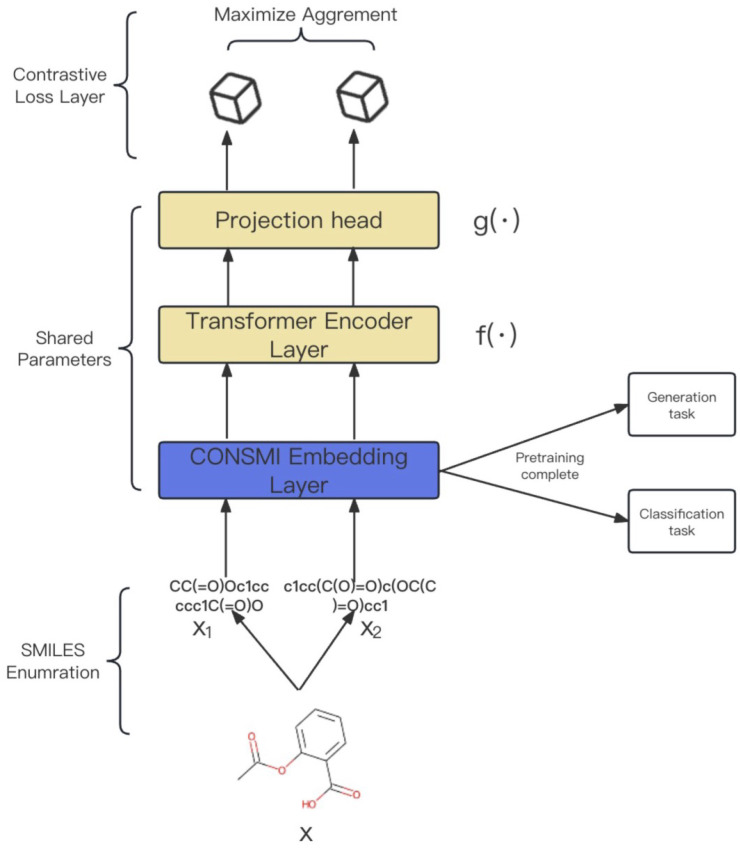
Overview of the CONSMI framework. Firstly, provide a molecule *x*. Obtain two SMILES representations representing $x_{1}$ and $x_{2}$ from *x*, and then input these together into the model. A transformer encoder module f(·) and a project head module g(·) are trained to maximize the agreement using a contrastive loss function. *f* includes several layers of transformer encoder layers, and *g* includes a multilayer perceptron (MLP).

Here, we describe a contrastive learning framework using SMILES enumeration to learn more comprehensive potential representations of SMILES. As shown in Figure 3, (i) We first use the SMILES enumeration strategy to generate multiple different representations of a molecule. (ii) We then use a CONSMI embedding layer, a transformer encoder module, and a projection head with shared parameters to encode the input representations into latent space. (iii) We finally introduce a contrastive loss layer to calculate the contrastive loss in a batch of samples. The idea is to maximize the similarity of different SMILES representation vectors for the same molecule, while keeping the SMILES vectors of different molecules away from each other. The different SMILES enumeration representations of molecules can be obtained using the cheminformatics library RDKit [49].

**Figure 4:**
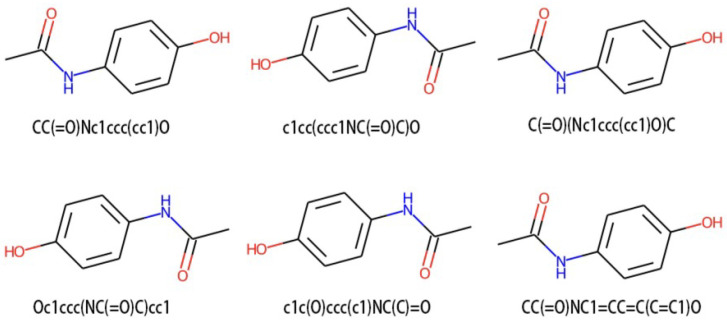
Visualization of different SMILES representations of the molecule acetaminophen, involving various transformations of its image. In principle, the DFS transversal graph that traverses all structures of acetaminophen has a certain degree of randomness. Since its images are generated according to specified rules by SMILES, each image is a different perspective from another images.

For each input molecule *x*, we perform SMILES enumeration to randomly select two types $x_{1}$ and $x_{2}$. SMILES enumeration is completed using a function from the chemical informatics library RDKit [49]. As Figure 4 shows, the atomic order of molecules is disrupted when converting to the molfile format, so RDKit is used to generate SMILES from the molecules in the molfile. Changing the dorandom parameter to true will randomize the DFS transversal graph when generating SMILES.

The function g(·) within the projection head component takes the latent representation from the preceding module and maps it into an embedding space denoted as *z*. To implement the function g(·), we have adopted a multilayer perceptron (MLP) architecture which consists of a single hidden layer with rectified linear units (ReLUs) as the activation function, followed by a linear output layer. This type of architecture is commonly utilized in neural networks.

Following Chen et al. [42], this work adopted the normalized temperature-scaled cross-entropy loss (NT-Xent) shown below as $l_{i,j}$

$$l_{i,j}=-log\frac{exp(sim\left(z_{i},z_{j}\right)/\tau )}{\sum_{k=1}^{2N}1_{k\neq i}exp\left(sim\left(z_{i},z_{k}\right)/\tau \right)}$$

where $sim\left(z_{i},z_{j}\right)=z_{i}^{T}z_{j}/\left|\right|z_{i}\left|||\right|z_{j}\left|\right|$ (i.e., cosine similarity) and $z_{i}$ and $z_{j}$ are a positive pair (i.e., $g(r_{i})$ and $g(r_{j})$ of the same molecule). The function $1_{k\neq i}$ is an indicator function equal to 1 if k≠i (i.e., the negative pairs) and τ denotes the temperature parameter. In addition, since each molecule generates two different SMILES representations, the mini-batch includes 2N examples, which means there are 2(N−1) negative examples and 2 positive examples for each sample.

### 3.2. Generation Module

**Figure 5:**
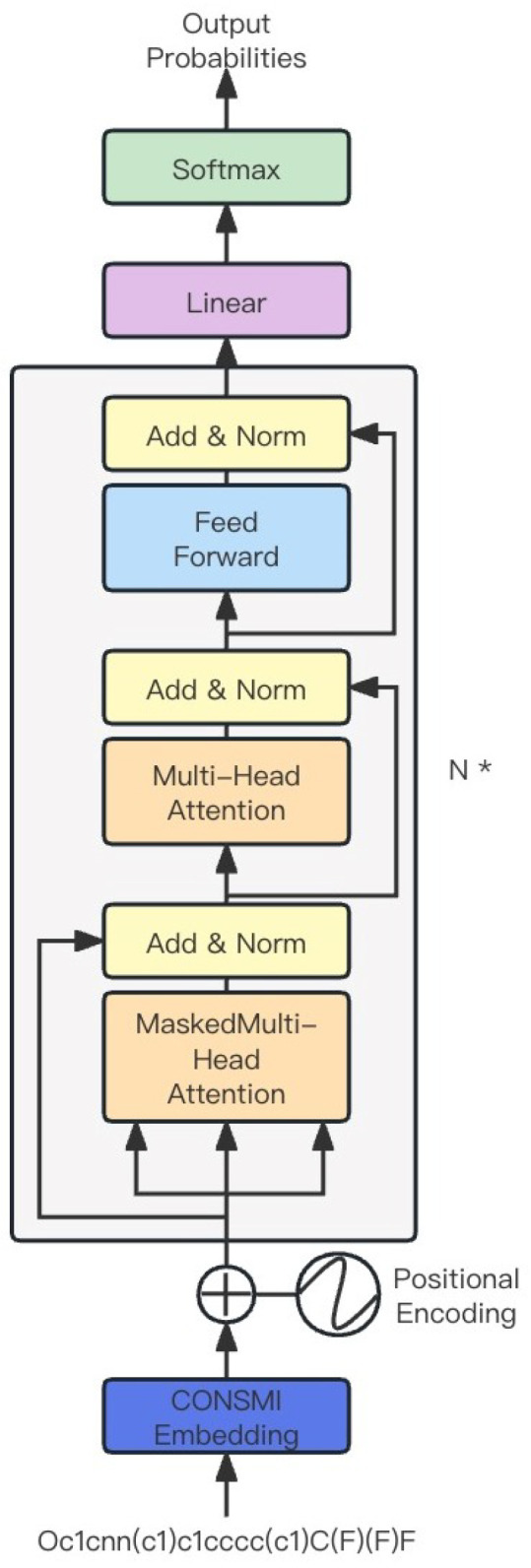
Overview of the generation module. N * means the module consists of a transformer decoder with N layers.

CON-GPT is composed of the generative pre-training transformer (GPT) model [54]. As shown in Figure 5, this module is built with N decoder blocks, where each block consists of a masked self-attention layer and a fully connected neural network. The self-attention layer produces a 256-dimensional vector, which serves as the input for the fully connected network. The hidden layer of the neural network generates a 1024-dimensional vector and applies the GELU (Gaussian error linear unit) activation function [55]. Its formula is as follows:

GELU(X)=x×P(X≤x)=x×ϕ(x),x∼N(0,1)

where *x* is the input value, while *X* is a Gaussian random variable with zero mean and unit variance. P(X≤x) is the probability that *X* is less than or equal to the given value *x*. The final layer of the fully connected network outputs a 256-dimensional vector, which is subsequently fed into the next decoder block. The module uses auto-regressive patterns for generation. We first use the CONSMI framework for pre-training, and then bring the pre-trained CONSMI embedding layer over for molecular generation. After introducing pre-trained CONSMI embedding, we used two fine-tuning methods: The first is to freeze the CONSMI embedding weights and only train additional modules. The other option is to not freeze the CONSMI embedding weights and train them together with additional modules.

### 3.3. Classifier Module

**Figure 6:**
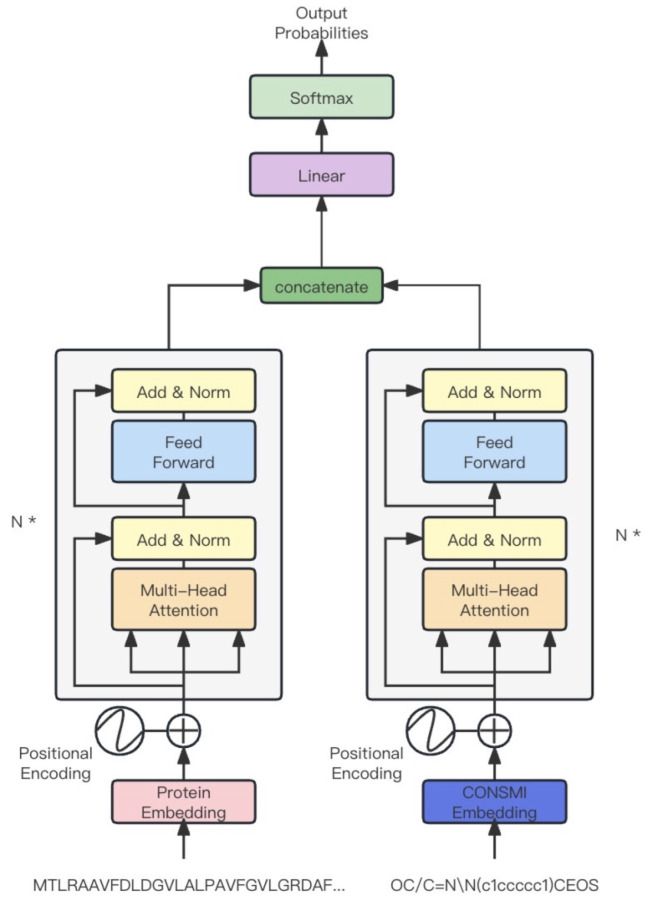
Overview of CON-UniT. N * means the module consists of two transformer encoders, each with N layers.

CON-UniT is based on the unified transformer [50]. As shown in Figure 6, this module consists of two transformer encoders, one encoding the protein sequence and the other encoding the SMILES representation sequence of the molecule. After being encoded by the encoder, they are concatenated and mapped to the binary dimensional space. The internal structure of the encoder is similar to the decoder structure used in the generation module.

## 4. Experiment Configuration

In this section, we first introduce two datasets for molecular generation experiments and datasets for the compound–protein interaction (CPI) task. Then, we describe the process and evaluation indicators of the experiment. Finally, we provide a detailed overview of the CONSMI training process for SMILES representation learning, the molecule generation task, and the CPI task.

### 4.1. Datasets

We selected SMILES representations with molecular weights between 250 and 350, a logP not exceeding 3.5, and a SMILES sequence length not exceeding 100 from the ZINC Clean Leads [56] to form our pre-training dataset. There was a total of 37,956,795 SMILES sequences. We divided the training and testing sets randomly at 19:1. We hoped to pre-train molecules on a large dataset and learn more comprehensive molecular representations. MOSES [57] is a set of lead-like molecules extracted from the Zinc dataset, and its distribution is very similar to that of ideal drug molecules. We used the MOSES dataset to generate new drug-like molecules. GuacaMol [58] is a subset of the database ChEMBL [59] which contains 1.6 million molecules. It was used to verify the migration ability of the model on different molecular distributions.

We also used the Celegans [60] and DAVIS [53] datasets for experiments on compound–protein interactions. Every dataset was further divided into three sets with a ratio of 8:1:1 for training, validating, and testing, respectively.

### 4.2. Evaluation Metrics

The primary objective of our model is to generate a diverse set of molecules. To assess the quality of the generated molecules, we employed five distinct 2D-level metrics: Validity, Uniqueness, Novelty, Success Rate, and Internal Diversity. These metrics were used to evaluate and compare the structural integrity, uniqueness, and ability to introduce new chemical structures of generated molecules, as well as their diversity in the chemical space.

**Validity** was determined by utilizing RDKit’s [49] molecular structure parser, which examines the valency of atoms and the consistency of bonds in aromatic rings. It assesses how accurately the generated molecules adhere to the rules and constraints of SMILES representations, ensuring proper atom connectivity and valence. Formally,

$$Validity=\frac{Number\;of\;valid\;SMILES}{Number\;of\;generated\;SMILES}$$

**Uniqueness** refers to the proportion of valid generated molecules that are distinct and not repetitive. A low uniqueness score indicates a higher frequency of duplicated or redundant molecules in the generated set. It reflects the model’s ability, or lack thereof, to learn a diverse distribution of molecules during the generation process. In experiments based on the MOSES benchmark, we computed Unique@K and for the first K 10,000 valid molecules in the generated set.

$$Uniqueness=\frac{Number\;of\;distinct,\;valid\;SMILES}{Number\;of\;Valid\;SMILES}$$

**Novelty** is defined as the proportion of generated molecules that do not exist in the training set. It measures the model’s capability to produce new and unseen molecules that were not encountered during the training process. A low novelty score suggests a higher likelihood of overfitting, where the model predominantly reproduces molecules already present in the training set rather than generating novel compounds.

$$Novelty=\frac{Number\;of\;novel\;SMILES\;not\;in\;training\;set}{Number\;of\;unique\;generated\;SMILES}$$

**Success Rate** is defined as the ratio of actual generation of available molecules, and from the perspective of unconditional generation of molecules, it should be the product of effectiveness, uniqueness, and novelty.

SuccessRate=Validity×Uniqueness×Novelty

**Internal Diversity ($IntDiv_{p}$)** was designed to measure the diversity of generated molecules and check for mode collapse or whether the model keeps generating similar structures. This involves calculating the mean power (*p*) of Tanimoto similarity (*T*) between fingerprints of all pairs of molecules (s1, s2) within the generated set (*S*).

$$IntDiv_{p}\left(S\right)=1-\sqrt[{p}]{\frac{1}{{\left|S\right|}^{2}}\sum_{s1,s2\in S}T{\left(s1,s2\right)}^{p}}$$

For the classification task, we employed Precision, Recall, and the F1 score as evaluation metrics.

**Precision** measures the accuracy of the model’s positive predictions. It is the proportion of correctly predicted positive samples out of all samples predicted as positive.

$$\mathrm{Precision}=\frac{TP}{TP+FP}$$

where TP is the number of positive instances correctly predicted as positive by the model and FP is the number of negative instances incorrectly predicted as positive by the model.

**Recall** measures the coverage of positive samples by the model. It is the proportion of correctly predicted positive samples out of all actual positive samples.

$$\mathrm{Recall}=\frac{TP}{TP+FN}$$

where FN is the number of positive instances incorrectly predicted as negative by the model.

**F1 Score** is the harmonic mean of precision and recall. It provides a comprehensive measure of a model’s performance by considering both precision and recall.

$$F1\;\mathrm{Score}=2\cdot \frac{\mathrm{Precision}\cdot \mathrm{Recall}}{\mathrm{Precision}+\mathrm{Recall}}$$

### 4.3. Training Details

The models were implemented based on Pytorch and trained on a GPU (Nvidia RTX3090) and a checkpoint was saved per epoch. We use the AdamW [61] optimizer, conducted a grid search in the interval of [0.0001, 0.01], and selected the value with the best performance in the validation set as the learning rate. We designated the target epoch as 100, and if there was no reduction in the loss of the validation set for 10 consecutive iterations, we saved the current model as the optimal one and concluded the training process. The batch size was 16, and the word vector dimension was 256. The parameters of the CON-GPT were consistent with those in MolGPT [35], so the data in the comparative experiment were directly used from the paper. We adopted the beam search procedure to generate multiple candidates. All generated candidates were canonicalized using RDkit and compared to the source molecules. The training settings for CON-UniT were the same as CONSMI, except that the batch size was changed to 128. The comparative data were collected from the paper on BCM-DTI [52].

## 5. Conclusions

In this work, we propose a contrastive learning pre-training framework called CONSMI, specifically designed for molecular SMILES representations. By leveraging SMILES enumeration as a data augmentation technique, we perform contrastive learning by using different SMILES representations of the same molecule as positive examples and different SMILES representations of different molecules as negative examples. The effectiveness of our framework is validated through experiments on the GPT model, showcasing its superior performance in molecular generation tasks.

The experimental results demonstrate that our pre-training framework significantly enhances the novelty and uniqueness of the generated molecules while maintaining a high level of validity. Our model is capable of generating more effective and novel molecules, while ensuring that their properties align with the distribution observed in the dataset. The evaluation conducted on both the MOSES dataset and the GuacaMol dataset further confirms the efficacy of our pre-training framework.

Moreover, our pre-training framework exhibits promising results in the compound–protein interaction task. The successful outcomes achieved across multiple datasets serve as further evidence that our framework facilitates the learning of improved molecular representations.

In summary, our proposed CONSMI framework contributes to the advancement of molecular generation tasks by enabling the generation of more effective and novel molecules while preserving their alignment with the dataset’s molecular distribution. Additionally, the framework demonstrates its efficacy in the compound–protein interaction task, showcasing its ability to facilitate more comprehensive molecular representation learning.

## Figures and Tables

**Table 1:** Comparison of the metrics for molecule generation using various approaches trained on the MOSES dataset.

Models	Validity	Unique@10k	Novelty	Success Rate	${\mathrm{IntDiv}}_{1}$
CharRNN	0.975	0.999	0.842	0.820	0.856
VAE	0.977	0.998	0.695	0.678	0.856
AAE	0.937	0.997	0.793	0.741	0.856
LatentGAN	0.897	0.997	0.949	0.849	0.857
JT-VAE	1.0	0.999	0.914	0.913	0.855
MolGPT	0.995	1.0	0.781	0.777	0.850
**CON-GPT (unfrozen)**	0.992	1.0	0.791	0.785	0.850
**CON-GPT (frozen)**	0.991	1.0	0.834	0.826	0.850

Our methods have been bolded.

**Table 2:** Comparison of the metrics for molecule generation using various approaches trained on the GuacaMol dataset.

Models	Validity	Unique	Novelty	Success Rate
SMILES LSTM	0.959	1.0	0.912	0.875
VAE	0.870	0.999	0.974	0.847
AAE	0.822	1.0	0.998	0.820
MolGPT	0.979	0.998	0.958	0.936
**CON-GPT (unfrozen)**	0.968	0.999	0.968	0.936
**CON-GPT (frozen)**	0.961	0.999	0.975	0.936

Our methods have been bolded.

**Table 3:** Example analysis of generated molecules: SMILES.

Number	Molecules Generated	Molecules in the Test Set
1	COc1ccc(NC(=O)N2CCN(C(=O)C3 CCCCC3)CC2)cc1	COc1ccc(NC(=O)N2CCN(C(=O)C3 CCCC3)CC2)cc1
2	Cc1nc2cc3c(cc2n1CC(=O)NC1CCCCC1) OCCO3	Cc1nc2cc3c(cc2n1CC(=O)NC1CCCC1) OCCO3
3	Cn1ccc(C(=O)Nc2cc(F)ccc2N2CCCC2)cc1=O	Cn1ccc(C(=O)Nc2cc(F)ccc2N2CCCCC2)cc1=O
4	Cc1cc(CN(C)C(=O)Nc2ccccc2N2CCCC2)no1	Cc1cc(CN(C)C(=O)Nc2ccccc2N2CCCCC2)no1
5	COc1ccccc1-c1noc(C(=O)N2CCCC2)c1N	COc1ccccc1-c1noc(C(=O)N2CCCCC2)c1N
6	CC(CC#N)N(C)C(=O)Nc1ccccc1N1CCCC1	CC(CC#N)N(C)C(=O)Nc1ccccc1N1CCCCC1

**Table 4:** Example analysis of generated molecules: molecular properties.

Number	Tanimoto Similarity	QED (Generated)	SAScore (Generated)	LogP (Generated)
1	0.958	0.916	1.839	2.952
2	0.957	0.940	2.318	2.565
3	0.957	0.941	2.184	2.377
4	0.956	0.950	2.102	3.247
5	0.952	0.935	2.284	2.168
6	0.952	0.925	2.685	3.053

**Table 5:** The impact of different τ values on generation tasks.

τ	0.05	0.10	0.50	1.00
**Loss**	0.16411	0.16326	0.16379	0.17299

**Table 6:** Experimental results of the compound–protein interaction classification task on the Celegans dataset.

Models	F1	Precision	Recall
GNN-CPI	0.933	0.938	0.929
TransformerCPI	0.952	0.952	0.953
Moltrans	0.954	0.947	0.962
BCM-DTI	0.969	0.967	**0.971**
**UniT**	0.964	0.966	0.961
**CON-UniT**	**0.969**	**0.972**	0.966

Top performed method in each metric is bold.

**Table 7:** Experimental results of the compound–protein interaction classification task on the DAVIS dataset.

Models	F1	Precision	Recall
GNN-CPI	0.658	0.647	0.669
TransformerCPI	0.584	0.46	0.8
Moltrans	0.306	0.185	**0.884**
BCM-DTI	0.611	0.853	0.476
**UniT**	0.841	0.844	0.837
**CON-UniT**	**0.868**	**0.874**	0.862

Top performed method in each metric is bold.

## Data Availability

The source code and datasets are available at https://github.com/WaterFlow1/CONSMI.git (accessed on 15 January 2024).
